# 

**Published:** 1862-12

**Authors:** 


### TO CORRESPONDENTS.

Many good communications are sent for the Journal. The editor writes all the articles which appear in its pages, except where his positions are illustrated by authentic facts of others well expressed; and even if it were not so, all articles would be rigidly excluded which reflect on private persons, on clergymen, on old-school physicians, or the administrators of the Government and its laws. If any body is particularly anxious to "blaze away" at either, it can be done to the greatest public good in the shape of a paid-for advertisement, in some extensively circulated newspaper, with the writer's full name, post-office address, street, and number.

### SUBSCRIBERS

Will please remember that their subscriptions end with this December number, and that the Journal will not be sent in any single case unless the desire is expressed in writing, accompanied with one dollar; hence, those who do not wish to continue need not be at the trouble of writing to that effect. Those who have failed to receive any number, will be supplied without charge, on application. Any back number from the first volume will be furnished for ten cents.

